# Congenital pulmonary airway malformation: A rare diagnosis in adulthood

**DOI:** 10.1002/rcr2.895

**Published:** 2021-12-20

**Authors:** Raquel Viana, Lina Carvalho, Cláudia Santos

**Affiliations:** ^1^ Pulmonology Department Centro Hospitalar de Leiria Leiria Portugal; ^2^ Anatomical Pathology Unit Centro Hospitalar Universitário de Coimbra Coimbra Portugal

**Keywords:** airway malformation, consolidation, cysts

## Abstract

This paper consists of a clinical image of a complex developmental anomaly that is usually diagnosed prenatally or during childhood. Its detection in adult life is very rare, as happened in the present case.

This paper consists of a clinical image of a complex developmental anomaly that is usually diagnosed prenatally or during childhood. Its detection in adult life is very rare, as happened in the present case.

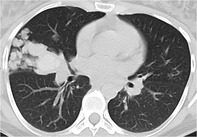

## CLINICAL IMAGE

**FIGURE 1:**
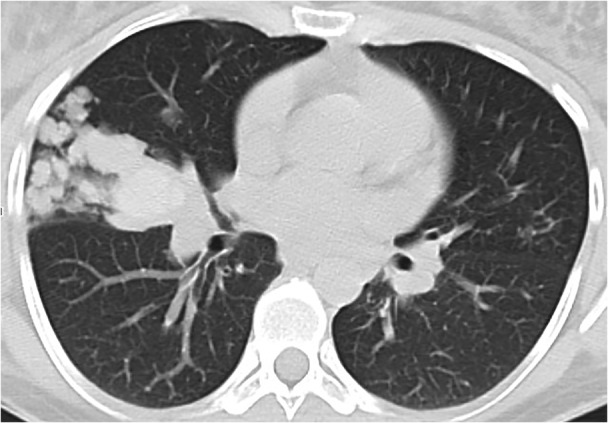
Middle lobe consolidation with polylobulated morphology on chest computed tomography

**FIGURE 2:**
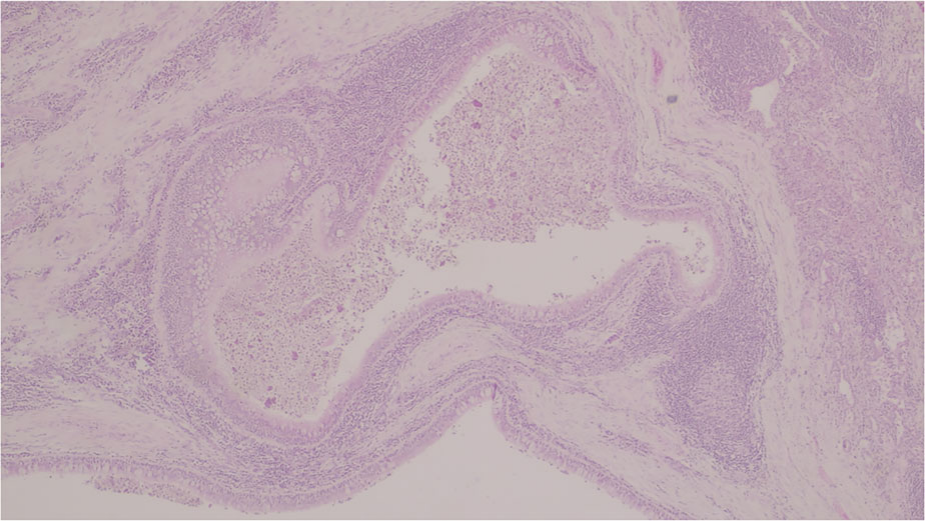
Typical cystic adenomatoid malformation (HE ×40)

A 28‐year‐old woman presented with chronic cough. She was a non‐smoker, with a history of asthma and an episode of community‐acquired pneumonia 1 year before. Computed tomography (CT) scan showed right apical and middle lobe polylobulated consolidations (Figure 1). Bronchial biopsies and CT‐guided transthoracic biopsy were inconclusive. No microorganisms were isolated and there were no signs of neoplastic process. Positron emission tomography‐CT did not exclude malignancy (18‐fluorodeoxyglucose positron emission tomography CT [18FDG PET/CT] maximum standardized uptake value [SUV_max_] = 2.4). Surgical biopsy was performed, demonstrating cavities covered with respiratory epithelium exhibiting mucinous cell hyperplasia, consistent with a type 1 congenital pulmonary airway malformation (CPAM) (Figure 2). Surgical removal of the lesion was the chosen treatment. The patient remained asymptomatic afterwards. CPAM is an uncommon developmental anomaly of unknown cause, characterized by overgrowth of terminal bronchioles and intercommunicating cysts. It is usually unilateral with involvement of a single lobe and most cases are recognized in the first years of life.^1^ Presentation in adulthood is extremely rare, and usually involves a history of recurrent pneumonia. CPAM may be confused with infection or neoplastic process, as in the present case, where the radiological presentation was atypical. Malignant transformation has been described, thus surgical resection is the gold‐standard treatment.^2^

## CONFLICT OF INTEREST

None declared.

## ETHICS STATEMENT

The authors declare that appropriate written informed consent was obtained for the publication of this manuscript and accompanying images.

## Data Availability

Data available on request from the authors.
